# Inhibition of the TLR/NF-*κ*B Signaling Pathway and Improvement of Autophagy Mediates Neuroprotective Effects of Plumbagin in Parkinson's Disease

**DOI:** 10.1155/2022/1837278

**Published:** 2022-12-22

**Authors:** Yan Su, Mao Li, Qi Wang, Xingfeng Xu, Peifang Qin, Haitao Huang, Yuting Zhang, Yali Zhou, Jianguo Yan

**Affiliations:** ^1^Department of Physiology, Guilin Medical University, Guilin, 541199 Guangxi, China; ^2^Guangxi Key Laboratory of Brain and Cognitive Neuroscience, Guilin Medical University, Guilin, 541199 Guangxi, China; ^3^Department of Microbiology, Guilin Medical University, Guilin, 541199 Guangxi, China

## Abstract

A naphthoquinone molecule known as plumbagin (PL), which has a wide range of pharmacological properties including antitumor, antioxidation, anti-inflammation, and neuroprotective effects, is extracted from the roots of the medicinal herb *Plumbago zeylanica* L. Plumbagin has been studied for its potential to treat Parkinson's disease (PD). However, its effectiveness and mechanism are still unknown. This study intends to evaluate plumbagin's effectiveness against PD *in vitro* and *in vivo*. Plumbagin partially repaired the loss of dopaminergic neurons in the nigral substantia nigra and the resulting behavioural impairment caused by MPTP or MPTP/probenecid in mice. Furthermore, plumbagin treatment significantly inhibited the TLR/NF-*κ*B pathways. It reduced the TNF-*α*, IL-6, and IL-1*β* mRNA expression in PD mice induced by MPTP or MPTP/probenecid, which was consistent with the findings in the inflammatory model of BV2 cells induced by MPP+ or LPS. In addition, plumbagin treatment enhanced the microtubule-associated protein 1 light chain 3 beta (LC3) LC3-II/LC3-I levels while decreasing the p-mTOR and p62 protein accumulation in PD mice induced by MPTP or MPTP/probenecid, which was similar to the results obtained from the experiments in SH-SY5Y and PC12 cells induced by MPP+. Consequently, our results support the hypothesis that plumbagin, by promoting autophagy and inhibiting the activation of the TLR/NF-*κ*B signaling pathway, is a promising treatment agent for treating Parkinson's disease (PD). However, to confirm plumbagin's anti-PD action more thoroughly, other animal and cell PD models must be used in future studies.

## 1. Introduction

Parkinson's disease (PD) is the second most common neurological disorder after Alzheimer's disease (AD). PD affects around 2% of those over the age of 65 years. People under the age of 40 have a minimal probability of developing PD [1]. People's health and quality of life are seriously damaged, especially as they get older. Lewy bodies (LBs), which are mainly comprised of *α*-synuclein, and the gradual degradation of dopaminergic neurons in the substantia nigra pars compacta (SNpc) are two of the main pathogenic aspects of Parkinson's disease (PD) [2–4]. Dopamine (DA) neuronal loss in the SNpc is the cause of PD symptoms such as tremors, bradykinesia, rigid muscles, speech and motor deficits, postural and balance abnormalities, and problems with automatic movements [5]. The pathogenesis of PD is complex and closely related to mitochondrial dysfunction, oxidative stress, neuroinflammation, abnormal aggregation of *α*-synuclein, and autophagy dysfunction [6–9]. Levodopa is frequently used in therapeutic intervention, although prolonged use might have substantial negative effects. Interestingly, levodopa cannot prevent neuron deterioration and only temporarily relieve the symptoms. Therefore, the development of novel prophylactic and therapeutic drugs is urgently required.

Numerous neurological and behavioural conditions, including AD, PD, and depression, are linked to neuroinflammation [10]. Neuroinflammation aggravates the dopaminergic neurons' loss in PD. The widespread expression of cytokines and chemokine receptors in dopaminergic neurons has raised the possibility that these cells respond to inflammatory mediators released by activated microglia [11]. Chronic neuroinflammation, which is mediated by neighbourhood microglia and, to a lesser extent, astrocytes and oligodendrocytes, causes neuronal death in PD patients [12]. Microglia are specialized immune cells that produce inflammatory mediators in response to tissue injury [13, 14]. In both familial and sporadic PD patients, there is a sudden increase in SNpc and microglia activation in the olfactory bulb [12]. Microglial activation in the SNpc olfactory bulb was dramatically elevated in familial and sporadic PD patients [12].

Autophagy's catabolic mechanism speeds up the destruction of excessive or otherwise defective proteins and organelles. Neurodegenerative disorders like PD are characterized by the accumulation of abnormal proteins and/or damaged organelles, and there is considerable evidence that autophagy dysregulation plays a role in this process [15]. Autopsy evidence revealed that autophagy markers are disrupted in several neurodegenerative diseases [16]. In a PD model, clearing accumulating proteins through autophagy is essential [17]. Abnormalities mostly cause PD in autophagy, and increasing autophagy is one treatment method [18].

Numerous medicinal herbs have a wide range, low toxicity, minimal side effects, and a variety of action targets. Recent studies have suggested that Chinese herbal medicine may contribute to treating and preventing PD. However, adopting Chinese herbal medicine for PD treatment and prevention has certain disadvantages. For instance, some active components of Chinese herbal medicine have low bioavailability, some have poor active ingredients and extraction efficiency, and some active ingredients are unstable and disintegrate easily. The structure of some active components of Chinese herbal medicine is unclear, which hinders the study and application of its mechanism. The research for Chinese herbal active components with high bioavailability, a large volume of extraction and synthesis, clear and stable active ingredients, diverse action targets, and efficient PD prevention and therapy has become increasingly important.

Plumbagin (PL) is a medicinal compound extracted from the *Plumbago zeylanica* L. plant's root [19]. Analysis of its molecular structure revealed it to be a naphthoquinone compound [20] that can be extracted and synthesized in large amounts. The bioactive ingredient is stable. It has antitumor [21, 22], antioxidation [23, 24], anti-inflammatory [25, 26], and neuroprotective [27, 28] pharmacological properties, but its ability to prevent or treat Parkinson's disease and its molecular mechanism are unexplored. We sought to determine whether plumbagin could protect dopaminergic neurons in mice administered MPTP poisoning because controlling autophagy and obstructing TLR/NF-*κ*B inflammatory pathways are potential approaches to preventing and treating PD. Additionally, the impact of plumbagin on the TLR/NF-*κ*B signaling pathway in the LPS-induced BV2 cells model and the autophagy pathway in the MPP+ -induced SH-SY5Y and PC12 cells PD model was assessed. Our present results might be applied to the therapeutic use of medicinal herbs for treating and preventing PD.

## 2. Materials and Methods

### 2.1. Chemical and Reagents

The plumbagin (purity >98%), MPTP, MPP+, probenecid, LPS, and *β*-actin antibody were purchased from Sigma Biotechnology Inc (MA, USA). Millipore Corporation (USA) provided the anti-tyrosine hydroxylase (TH) antibody. The primary antibodies for TLR2, TLR4, and NF-*κ*B p65 were obtained from Affinity Biosciences Ltd (OH, USA). Cell Signaling Technology (Shanghai, China) supplied the LC3B and p62 antibodies. The p-mTOR antibody utilized in this work was obtained from Santa Cruz Biotechnology Inc (TX, USA). The horse serum, MEM medium, Ham's F-12 medium, sodium pyruvate, MEM nonessential amino acids, and glutamine additive were all delivered by Gibco (Thermo Fisher Scientific, USA). The ShuangRu Biotechnology Co., Ltd (Shanghai, China) provided the fetal bovine serum.

### 2.2. Animals

To conduct this study, we used male C57/6J mice that we obtained from Hunan Silaike Jingda Laboratory Animal Co. Ltd (China). Every cage had enough space for five animals. They were kept at room temperature (23 ± 2°C) with a relative humidity of 60 ± 10% and a 12-hour light-dark cycle, with the food, water, and bedding materials being rotated regularly. One week of preadministration acclimatization was permitted.

### 2.3. Subacute PD Mouse Model Induced by MPTP

Three-month-old male C57BL/6 mice were randomly assigned to one of four groups (*n* = 15 mice/group): control, MPTP, PL+MPTP, or PL. Mice in the control and MPTP groups received the vehicle intragastrically, while mice in the PL and PL+MPTP groups received 5 mg/kg of PL (Sigma-Aldrich, USA) for 14 days. From day 15, after 1 h of intragastric gavage, MPTP (Sigma-Aldrich, USA) was intraperitoneally injected in the MPTP and PL+MPTP groups mice at a dose of 25 mg/kg. In contrast, normal saline was intraperitoneally injected in the control and PL groups for 7 days. From day 22, each group was administered via gavage for another 14 days. Before the mice were put down and their brains were removed for immunofluorescence and Western blotting, they underwent several behavioural assessments.

### 2.4. MPTP/Probenecid Chronic PD Mouse Model

For this study, 60 male C57BL/6 mice aged 3 months were split into 4 groups (*n* = 15 per group): control, MPTP/p, PL+MPTP/p, and PL. For 14 days, mice in the PL and PL+MPTP/p groups received intragastrical injections of 5 mg/kg of PL (Sigma-Aldrich). In contrast, mice in the control and MPTP groups received intragastrical administrations of the vehicle. Mice in the MPTP/p and PL+MPTP/p groups received 25 mg/kg MPTP (Sigma-Aldrich) subcutaneously for 30 minutes after receiving 250 mg/kg probenecid (Sigma-Aldrich) intragastrically on day 15. At the same time, mice in the control and PL groups received normal saline intraperitoneally 10 times for five weeks. In addition, PL and vehicle were both administered intranasally for a total of 49 days. Moreover, PL and vehicle were given intranasally for 49 days. Following the behavioural examination, the mice were slaughtered, and their brains were taken for immunofluorescence and Western blotting.

### 2.5. Rotarod Test

The mice were trained for two days on the rotarod (Xin Run, Shang Hai) at 10 rpm three times a day (at 30-minute intervals). Each mouse's ability to remain on the rotarod for a long time while the speed climbed from 0 to 30 revolutions per minute was noted. The last test result was the average latency to fall off the rod, which was determined by doing 5 separate experiments.

### 2.6. Open Field Test

The mice spent 20 minutes roaming freely in a 50 × 50 × 25 cm open field box (Xin Run, Shang Hai). With the help of the Supermaze system's built-in camera, we were able to track the mice's every step and calculate their total distance travelled. The box was wiped down with 75% alcohol, and the mouse' face and urine were removed after each test to ensure no lingering odour would affect the next mouse' performance.

### 2.7. Immunofluorescence Staining

The substantia nigra of mice brains was sectioned and frozen before being treated with anti-TH (1 : 250, Millipore) at 4° C for one night. The slices were washed and then incubated with a secondary antibody (IgG (H+L)) for 1 hour at room temperature. The Olympus microscope was used to capture the images. We used ImageJ software to quantify the number of times TH-positive dopaminergic neurons were detected in the SNpc region.

### 2.8. Real-Time PCR Analysis

TRIzol (Invitrogen, USA) was used to extract total RNA from the substantia nigra of mice brains and BV2 cells. cDNA was synthesized with the help of the cDNA Synthesis Kit (Monad). Amplification was performed using Thermal Cyclers (Thermo Fischer Scientific, USA) using the cDNA as templates for real-time PCR using a Real-Time PCR Reagents Kit (Monad Biotech Co., Ltd, Wuhan, China). The real-time PCR primers are listed in Table 1.

### 2.9. Western Blotting

Nuclear and cytoplasmic protein was isolated from mouse brain substantia nigra and cell using the Beyotime Nuclear and Cytoplasmic Protein Extraction Kit and RIPA buffer (Beyotime). The NanoDrop 2000 was used to quantify protein concentrations (Thermo Fischer Scientific, USA). Electrophoretically, protein extracts were separated on sodium dodecyl sulfate-polyacrylamide (SDS-PAGE) gels before being transferred to a PVDF membrane (Merck Millipore, Germany). Primary antibodies were incubated at 4°C overnight after being blocked in 5% BSA in the TBST buffer for 1 h at room temperature. Anti-actin (1 : 5000), anti-LC3B (1 : 1000), anti-p62 (1 : 1000), anti-pmTOR (1 : 3000), anti-TLR2 (1 : 500), anti-TLR4 (1 : 500), anti-NF-kappa B p65 (1 : 500), and anti-histone H3 (1 : 500) primary antibodies were diluted as described. The PVDF membrane was incubated with HRP-labeled IgG antibody (1 : 10000) in TBST for 2 hours at room temperature after being washed three times in TBST for 30 minutes. Quantitative studies were performed using the ImageJ program and the enhanced chemiluminescence technique (Bio-Rad).

### 2.10. Cell Culture

Cell lines from SH-SY5Y cells, PC12 cells from rat adrenal pheochromocytomas, and BV2 cells from mouse microglia were obtained from the National Collection of Authenticated Cell Cultures. Medium Eagle's Medium (MEM) was used to culture SH-SY5Y cells, and 10% FBS, Ham's F-12, sodium pyruvate solution, MEM nonessential amino acid solution, glutamine additive, and 0.1% of 100 IU/mL penicillin-streptomycin combination were added to the medium. PC12 cells were cultured in RPMI-1640 media with 5% fetal bovine serum, 10% horse serum, and 0.1% of 100 IU/mL penicillin-streptomycin. DMEM high-glucose medium with 10% FBS and 0.1% of 100 IU/mL penicillin-streptomycin combination was used to cultivate BV2 cells. We used a 37°C incubator with a 5% CO_2_ environment to cultivate the cells.

### 2.11. Cell Viability Assay

We examined plumbagin's potential to suppress MPP+-induced cell apoptosis in PC12 and SH-SY5Y cells using the cell counting kit-8 (CCK-8) assay. In a 96-well plate, 8 × 10^3^ PC12 and SH-SY5Y cells were seeded per well. The cells were treated with 0, 0.001, 0.01, and 0.1 *μ*M plumbagin for 24 hours. Afterwards, 500 *μ*M MPP+ was added to the plumbagin incubation for 24 hours. After adding CCK-8 reagents (Dojindo), we left the mixture to incubate at 37°C for 4 hours on the move. A microplate reader (Bio-Rad) was used to determine the absorbance at 450 nm.

### 2.12. Statistical Analyses

The data was analyzed using SPSS 22.0 and GraphPad Prism 8.0. The results were expressed as a mean ± SEM. Tests of significance and one-way analysis of variance (ANOVA) were used to examine the data. The *P* value of < 0.05 was considered statistically significant.

## 3. Results

### 3.1. Plumbagin Reduces Behavioural Abnormalities and Decreases DA Neuron Loss in MPTP- or MPTP/Probenecid-Induced PD Mice

**Figure 1:**
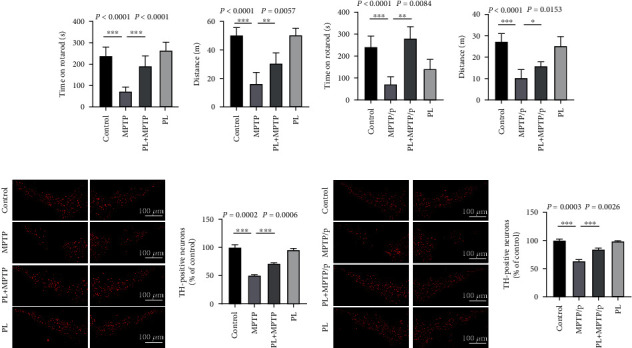
Plumbagin improves behavioural disorders and suppresses the loss of DA neurons in PD mice induced by MPTP or MPTP/probenecid. (a) The open field and (b) rotarod tests were performed to detect motor deficits in each mice group with the vehicle, MPTP, plumbagin+MPTP, and plumbagin (*n* = 15). (c) The open field and (d) rotarod tests were applied to detect motor deficits in each mice group with the vehicle, MPTP/p, plumbagin+MPTP/p, and plumbagin (*n* = 15). (e) Immunofluorescence staining was performed to detect TH-positive neurons in the SNpc of each mice group with the vehicle, MPTP, plumbagin+MPTP, and plumbagin (*n* = 3). Scale bar = 100 *μ*m. (f) Quantitative data of TH-positive neurons in SNpc of each mice group with the vehicle, MPTP, plumbagin+MPTP, and plumbagin (*n* = 3). (g) Immunofluorescence staining was performed to detect TH-positive neurons in the SNpc of each mice group with the vehicle, MPTP/p, plumbagin+MPTP/p, and plumbagin (*n* = 3). Scale bar = 100 *μ*m. (h) Quantitative data of TH-positive neurons in SNpc of each mice group with the vehicle, MPTP/p, plumbagin+MPTP/p, and plumbagin (*n* = 3). Data is presented as the mean ± SEM, ^∗∗∗^*P* < 0.001, ^∗∗^*P* < 0.01, and ^∗^*P* < 0.05.

In order to ensure the successful establishment of the PD model, we also identified it by the expression of *α*-synuclein protein by Western blotting. Compared with the control group, *α*-synuclein protein increased significantly after MPTP or MPTP/probenecid induction (Figure S1). Mouse motor ability is often assessed using either the rotarod test or the open field test, examples of standard behavioural studies. The rotarod and open field tests showed that MPTP-induced PD animals had considerably shorter latency times and distances than control mice. But therapy with plumbagin helped reduce the severity of the behavioural issue (Figures 1(a) and 1(b)). We investigated the possible protective impact of plumbagin on DA neurons since their demise is associated with PD. Dopamine (DA) neurons in the substantia nigra are greatly reduced in number in MPTP-induced PD animals. While DA neurons are lost in MPTP-induced PD rats, we found that plumbagin protects these cells (Figures 1(e) and 1(f)). Compared to their acute counterparts, chronic MPTP-induced PD animal models reveal a moderate but progressive reduction in dopaminergic neurons. This is similar to the degenerative process of PD, in which DA neurons are gradually destroyed. Thus, we used MPTP and probenecid to investigate the chronic PD mouse model. We evaluated the efficacy of plumbagin in these mice after inducing a long-term animal model of PD using MPTP and probenecid. Figures 1(c) and 1(d) show that plumbagin reduced behavioural impairment in PD mice and prevented the loss of DA neurons (Figures 1(g) and 1(h)). These findings suggest that plumbagin can be utilized as a treatment for PD since it reduces symptoms similar to PD.

### 3.2. Plumbagin Inhibited MPTP or MPTP/Probenecid-Induced TLR/NF‐*κ*B Activation in PD Mice

**Figure 2:**
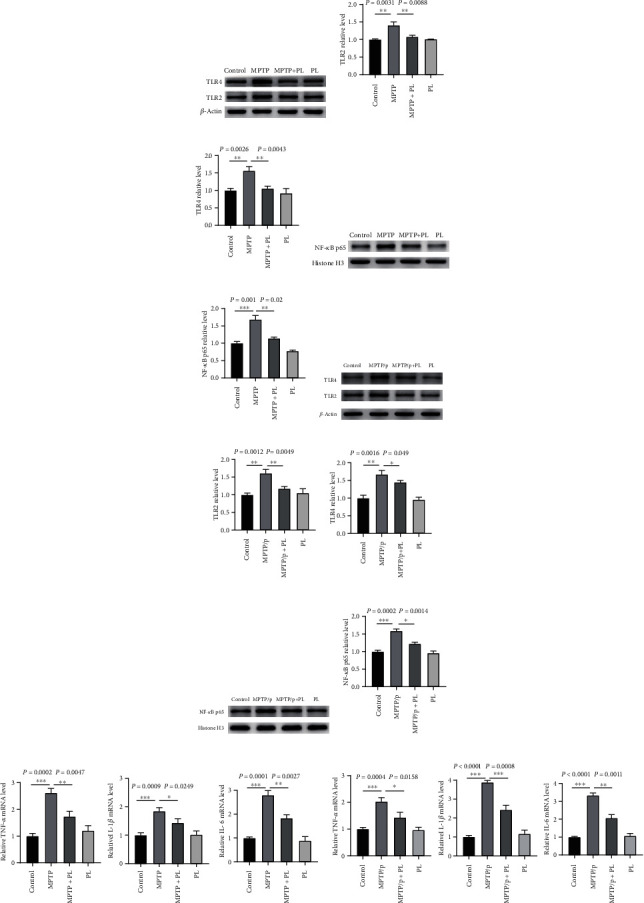
Plumbagin suppressed TLR/NF-*κ*B activation in PD mice induced by MPTP or MPTP/probenecid. (a) Western blotting analysis of TLR2 and TLR4 in mice treated with the vehicle, MPTP, plumbagin+MPTP, and plumbagin. (b) Quantitative data of the TLR2 protein levels in each mice group. (c) Quantitative data of the TLR4 protein levels in each mice group. (d) Western blotting analysis of NF-*κ*B p65 in mice treated with the vehicle, MPTP, plumbagin+MPTP, and plumbagin. (e) Quantitative data of the NF-*κ*B p65 protein levels in each mice group. (f) Western blotting analysis of TLR2 and TLR4 in mice treated with the vehicle, MPTP/probenecid, plumbagin+MPTP/probenecid, and plumbagin. (g) Quantitative data of the TLR2 protein levels in each mice group. (h) Quantitative data of the TLR4 protein levels in each mice group. (i) Western blotting analysis of NF-*κ*B p65 in mice treated with the vehicle, MPTP, plumbagin+MPTP, and plumbagin. (j) Quantitative data of the NF-*κ*B p65 protein levels in each mice group. (k) The quantification of the mRNA levels of TNF-*α*, IL-1*β*, and IL-6 in SNpc of mice treated with the vehicle, MPTP, plumbagin+MPTP, and plumbagin was examined by RT-qPCR (*n* = 3). (l) The quantification of the mRNA levels of TNF-*α*, IL-1*β*, and IL-6 in the SNpc of mice treated with the vehicle, MPTP/p, plumbagin+MPTP/p, and plumbagin examined by RT-qPCR (*n* = 3). Data is expressed as the mean ± SEM, ^∗∗∗^*P* < 0.001, ^∗∗^*P* < 0.01, and ^∗^*P* < 0.05.

Brains of many damaged animal models produce inflammatory mediators such as TNF-*α*, IL-1*β*, and IL-6, all of which are regulated by pattern recognition receptors called Toll-like receptors (TLRs). TLR2 and TLR4 expressions were significantly higher in MPTP-induced PD model mice compared to the control group, based on nuclear translocation of the nuclear factor kappa B p65 protein (Figures 2(a)–2(e)). The treatment of MPTP-induced PD mouse models with plumbagin dramatically suppressed the expression of TLR2 and TLR4 proteins and the nuclear translocation of NF-*κ*B p65 protein. Previous research has shown that plumbagin may significantly reduce inflammation in the substantia nigra of PD mouse models (Figures 2(a)–2(e)). Proinflammatory cytokines, such as TNF-*α*, IL-1*β*, and IL-6, stimulate the inflammatory cascade and are linked to the worsening or development of PD; the NF-*κ*B signaling pathway regulates their expression.

Plumbagin's effects on the mRNA expression of inflammatory factors TNF-*α*, IL-1*β*, and IL-6 were examined in the substantia nigra of MPTP-induced PD animals. Increased levels of TNF-*α*, IL-1*β*, and IL-6 mRNA expression were seen in MPTP-induced PD animals, and their elevation was partly attenuated by plumbagin administration, as determined by real-time PCR (Figure 2(k)). In chronic PD mice produced by MPTP and probenecid, plumbagin decreased TLR2, and TLR4 protein expressions blocked the nuclear translocation of NF-*κ*B p65 protein (Figures 2(f)–2(j)) and suppressed the production of TNF-*α*, IL-1*β*, and IL-6 mRNA (Figure 2(l)). We also investigated how the protein *α*-synuclein, a biomarker of neuroinflammation in PD, is expressed. When MPTP or MPTP/probenecid was induced, *α*-synuclein protein considerably increased compared to the control group, and plumbagin therapy reversed the effects of MPTP or MPTP/probenecid (Figure S2).

### 3.3. Plumbagin Inhibited MPP+ or LPS-Induced TLR/NF-*κ*B Activation in BV2 Cells

**Figure 3:**
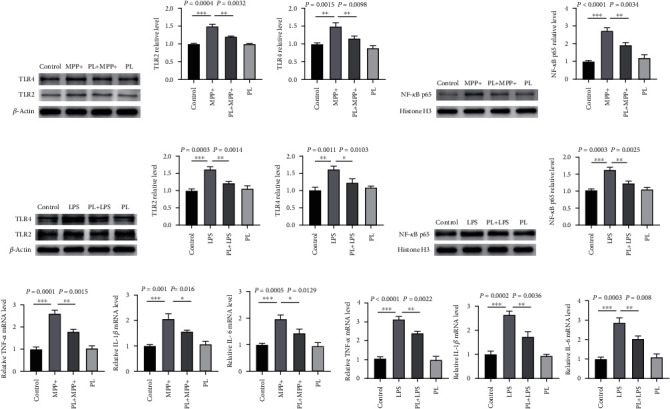
Plumbagin suppressed the activation of the TLR/NF-*κ*B signaling pathway in BV2 cells induced by MPP+ or LPS. (a) Western blotting analysis of TLR2 and TLR4 in the BV2 cells treated with vehicle, MPP+, PL+ MPP+, and PL. (b) Quantitative data of the TLR2 protein levels in the BV2 cells treated with vehicle, MPP+, PL+ MPP+, and PL. (c) Quantitative data of the TLR4 protein levels in the BV2 cells treated with vehicle, MPP+, PL+ MPP+, and PL. (d) Western blotting analysis of NF-*κ*B p65 in the BV2 cells with the vehicle, MPP+, PL+ MPP+, and PL. (e) Quantitative data of the NF-*κ*B p65 protein levels in the BV2 cells treated with vehicle, MPP+, PL+ MPP+, and PL. (f) Western blotting analysis of TLR2 and TLR4 in the BV2 cells treated with vehicle, LPS, PL+ LPS, and PL. (g) Quantitative data of the TLR2 protein levels in the BV2 cells treated with vehicle, LPS, PL+ LPS, and PL. (h) Quantitative data of the TLR4 protein levels in the BV2 cells treated with vehicle, LPS, PL+ LPS, and PL. (i) Western blotting analysis of NF-*κ*B p65 in the BV2 cells treated with vehicle, LPS, PL+ LPS, and PL. (j) Quantitative data of the NF-*κ*B p65 protein levels in the BV2 cells treated with vehicle, LPS, PL+ LPS, and PL. (k) The quantification of the mRNA levels of TNF-*α*, IL-1*β*, and IL-6 in the BV2 cells treated with vehicle, MPP+, PL+ MPP+, and PL was examined by RT-qPCR (*n* = 3). (l) The quantification of the mRNA levels of TNF-*α*, IL-1*β*, and IL-6 in the BV2 cells treated with vehicle, LPS, PL+ LPS, and PL was examined by RT-qPCR (*n* = 3). Data is expressed as the mean ± SEM, ^∗∗∗^*P* < 0.001, ^∗∗^*P* < 0.01, and ^∗^*P* < 0.05.

The molecular mechanism of PD was studied by conducting cell studies. TLR2, TLR4, and NF-*κ*B p65 protein expressions were measured in inflammatory models of BV2 cells triggered by MPP+ or LPS using Western blotting. TLR2 and TLR4 protein expressions and NF-*κ*B p65 nuclear translocation were considerably upregulated in BV2 cells after stimulation with MPP+ or LPS compared to the control group. In contrast, plumbagin administration reduced the expression of TLR2 and TLR4 proteins and dramatically prevented the nuclear translocation of NF-*κ*B p65 protein in BV2 cells triggered by MPP+ or LPS (Figures 3(a)–3(j)). After showing that plumbagin may reduce inflammation caused by MPTP and MPTP/probenecid in PD mice, we looked into its influence on inflammatory cytokines in BV2 cells caused by MPP+ or LPS. The real-time PCR analysis showed that the mRNA expression levels of TNF-*α*, IL-1*β*, and IL-6 in BV2 cells rose in response to MPP+ or LPS stimulation. In contrast, treatment with plumbagin somewhat attenuated the increase (Figures 3(k) and 3(l)). These results implied that plumbagin could effectively inhibit the inflammatory response induced by MPP+ or LPS in BV2 cells.

### 3.4. Plumbagin Increased Autophagy in MPTP- or MPTP/Probenecid-Induced PD Mice

**Figure 4:**
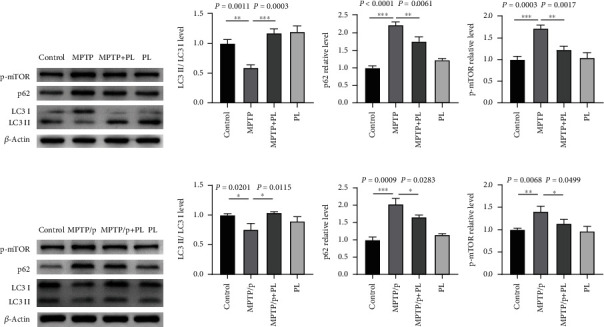
Plumbagin improved autophagy in PD mice induced by MPTP or MPTP/probenecid. (a) Western blotting analysis of LC3, p62, and p-mTOR in mice treated with the vehicle, MPTP, plumbagin+MPTP, and plumbagin. (b) Quantitative data of the LC3-II/LC3-I, p62, and p-mTOR protein levels in mice treated with the vehicle, MPTP, plumbagin+MPTP, and plumbagin. (c) Western blotting analysis of LC3, p62, and p-mTOR in mice treated with the vehicle, MPTP/p, plumbagin+MPTP/p, and plumbagin. (d) Quantitative data of the LC3-II/LC3-I, p62, and p-mTOR protein levels in mice treated with the vehicle, MPTP/p, plumbagin+MPTP/p, and plumbagin. Data are presented as the mean ± SEM (*n* = 3). ^∗∗∗^*P* < 0.001, ^∗∗^*P* < 0.01, and ^∗^*P* < 0.05.

Two major cellular processes affect multiple neurological illnesses: neuroinflammation and autophagy. Recent studies have demonstrated that regulating microglia activation via mitochondrial autophagy can increase neuronal survival in PD. Because it reduces neuroinflammation, autophagy also significantly positively affects ischemia [29]. Decreased autophagy is thought to have a role in neurodegenerative processes linked to chronic inflammatory states [30]. As a result, we further investigated plumbagin's effect on autophagy in MPTP-induced PD mice. Compared to the control group, MPTP-induced PD mice had higher levels of the autophagy substrate proteins p62 and p-mTOR, while LC3-II/LC3-I levels were lower. However, plumbagin treatment overcame this (Figures 4(a) and 4(b)). Additionally, we observed that plumbagin therapy reduced the ratio of LC3-II/LC3-I and the expression levels of autophagy substrate p62 and p-mTOR proteins in chronic PD rats caused by MPTP and probenecid (Figures 4(c) and 4(d)). Based on these results, plumbagin was demonstrated to improve autophagy in PD mice and enhance the clearance of autophagy substrates.

### 3.5. Plumbagin Improved Autophagy in the PD Models of SH-SY5Y and PC12 Cells Induced by MPP+

**Figure 5:**
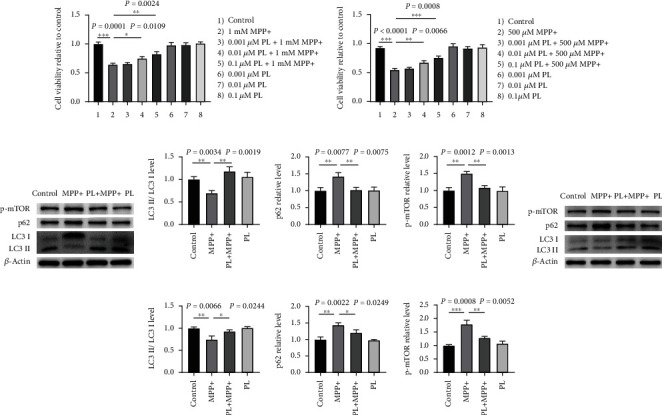
Plumbagin improved autophagy in the PD models of SH-SY5Y and PC12 cells induced by MPP+. (a) The effects of different doses of plumbagin on cell viability in SH-SY5Y cells. SH-SY5Y cells were pretreated with vehicle or plumbagin (0.001 *μ*M, 0.01 *μ*M, and 0.1 *μ*M) for 24 h, after which MPP+ (1 mM) was added to the cell culture plate, and the mixture was incubated for another 24 h. Cell viability was assessed by the CCK-8 assays. (b) The effects of different doses of plumbagin on cell viability in PC12 cells. PC12 cells were pretreated with vehicle or plumbagin (0.001 *μ*M, 0.01 *μ*M, and 0.1 *μ*M) for 24 h, after which MPP+ (500 *μ*M) was added to the cell culture plate, and the mixture was incubated for another 24 h. Cell viability was assessed by using the CCK-8 assay. (c) Western blotting analysis of LC3, p62, and p-mTOR in the SH-SY5Y cells treated with vehicle, MPP+, PL+ MPP+, and PL. (d) Quantitative data of the LC3-II/LC3-I, p62, and p-mTOR protein levels in the SH-SY5Y cells treated with the vehicle, MPP+, PL+ MPP+, and PL. (e) Western blotting analysis of LC3, p62, and p-mTOR in the PC12 cells-induced treatment with the vehicle, MPP+, PL+ MPP+, and PL. (f) Quantitative data of the LC3-II/LC3-I, p62, and p-mTOR protein levels in the PC12 cells treated with the vehicle, MPP+, PL+ MPP+, and PL. Data are expressed as the mean ± SEM (*n* = 3). ^∗∗∗^*P* < 0.001, ^∗∗^*P* < 0.01, and ^∗^*P* < 0.05.

Improving autophagy is a possible therapeutic option for PD, and its deficit in neurodegenerative illnesses is well established. Consequently, we investigated the PD's molecular mechanisms using cell experiments. Before being exposed to 1 mM and 500 *μ*M MPP+ for the same time in the SH-SY5Y and PC12 cells, plumbagin was pretreated on the cells for 0, 0.001, 0.01, and 0.1 *μ*M on SH-SY5Y and PC12 cells for 24 hours. There was a statistically significant decrease in cell viability compared to the control group after MPP+ treatment. The MPP+-induced drop-in survival rate in SH-SY5Y and PC12 cells were blocked at 0.1 *μ*M (Figures 5(a) and 5(b)). Subsequently, using MPP+ to induce PD in SH-SY5Y and PC12 cells, we examined how plumbagin affected autophagy in these cells. In MPP+-induced PD models of SH-SY5Y and PC12 cells, levels of the autophagy substrate p62 and the p-mTOR protein were elevated, but LC3-II/LC3-I levels were decreased. In contrast, plumbagin administration decreased the expression of the autophagy substrate p62 and the p-mTOR protein while increasing the levels of LC3-II/LC3-I (Figures 5(c)–5(f)).

### 3.6. Depiction of the Mechanism of Plumbagin against PD through Anti-Inflammatory and Autophagy Regulation

**Figure 6:**
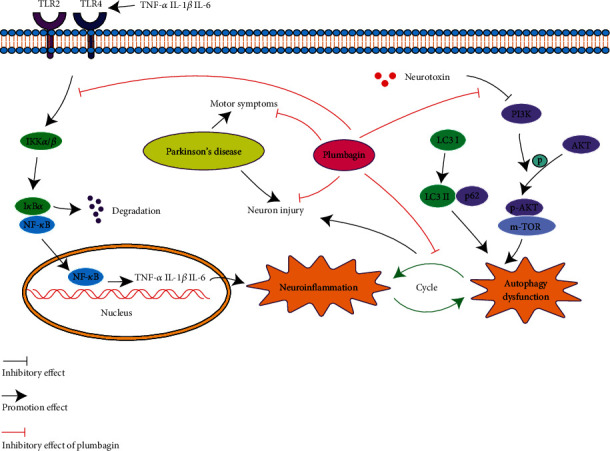
Depiction of the mechanism of plumbagin against Parkinson's disease through anti-inflammatory and autophagy regulation.

Multiple factors and mechanisms contribute to the pathogenesis of PD. These mechanisms interact and influence each other, posing several challenges to PD's research and treatment. Neuroinflammation and autophagy disturbances are both important factors in PD. Neuroinflammation leads to the pathogenesis of PD and the disturbance of autophagy, which aggravates neuroinflammation. Our study demonstrated that plumbagin possesses antineuroinflammatory and autophagy-improving properties (Figure 6).

## 4. Discussion

Neuroinflammation has been observed in many PD models, including neurotoxin-based models (such as 6-hydroxydopamine and MPTP) and *α*-synuclein-based models (such as *α*-synuclein transgenic animals, *α*-synuclein-based viral transfection models, and misfolded *α*-synuclein fibril administration models) [31]. Although the root cause of neuronal loss is unknown, autopsy studies have shown that the pathophysiology of PD is characterized by the substantia nigra inflammation defined by persistent and excessive microglia and astrocyte proliferation [32]. According to some research, inflammation and immunological responses are disease markers and pathogenic factors in the aetiology of familial and sporadic Parkinson's disease. Patients with Parkinson's disease have active microglia in the SNpc and putamen [33]. Tumor necrosis factor-alpha (TNF-*α*), interleukin (IL-1*β*), interleukin-6 (IL-6), chemokines, and active nitrogen (such as NO) and ROS are released by activated microglia, which can cause or aggravate the initiation of PD [34, 35]. Nuclear factor erythroid 2-related factor 2 (Nrf2) is a transcription factor that is a major regulator of endogenous antioxidant responses and can inhibit NF-*κ*B activity. Without Nrf2, the NF-*κ*B inhibitor (I*κ*B) is rapidly degraded by the proteasome, thereby increasing the NF-*κ*B levels [36, 37]. Studies have shown that plumbagin improves memory dysfunction in AD mice through the Nrf2/ARE pathway [38]. Nrf2 activators are a potential therapy for Parkinson's disease [39].

Unlike astrocytes and neurons, microglia have several Toll-like receptors (TLRs), which are a type of pattern recognition receptor. In the brains of animal models with diverse lesions, they can control NF-*κ*B and take part in distinct inflammatory responses, generating TNF-*α*, IL-1*β*, and IL-6. Through their ability to activate glial cells and other inflammatory factors, TLR2 and TLR4 are to blame for brain injury-related inflammation. There was an increase in both TLR2 and TLR4 expressions in the blood and brain of people with PD [40, 41]. NF-*κ*B has a critical role in regulating neuroinflammation. During quiescence, NF-*κ*B heterodimers (p50 and p65) are inactive in the cytoplasm, coupled to protein *κ*B inhibitors (I*κ*B). In contrast, NF-*κ*B is activated and initiates transcription of downstream target genes such as TNF-*α*, IL-1*β*, and IL-6 [42–44] when I*κ*B*α* is phosphorylated and degraded, allowing the NF-*κ*B dimer p50 and p65 to enter the nucleus. Therefore, indicators of NF-*κ*B activation include nuclear p65 expression and the generation of inflammatory cytokines.

Plumbagin exerts an antineuroinflammatory effect by reducing NF-*κ*B levels, downregulating the expression of iNOS and COX-2, and increasing the expression of Nrf2 and HO-1 on neuropathic pain induced by chronic compression injury in SD rats. Plumbagin inhibits chronic periodontitis by downregulating the TNF-*α*, IL-1*β*, and IL-6 expressions [28, 45]. In rats, it can also inhibit neuronal apoptosis and intimal hyperplasia following cerebral ischemia, as well as the inflammatory response induced by the TNF-*α*/NF-*κ*B pathway [46]. According to these investigations, plumbagin inhibited NF-*κ*B activation and downregulated the expression of inflammatory factors. Our research showed that plumbagin inhibited inflammation via the TLR/NF-*κ*B pathway, decreased TNF-*α*, IL-1*β*, and IL-6 mRNA expressions, and provided neuroprotection against subacute and chronic PD mice models. Similarly, plumbagin had the same effect in the inflammatory model of BV2 cells induced by MPP+ or LPS. There is currently no cure for Parkinson's disease, but research on neuron inflammation in the disease is advancing rapidly and could lead to a novel neuroprotective therapy strategy [31].

Numerous intracellular protein aggregates can cause mutations in neurodegenerative disorders, affecting autophagy and changing substrate clearance [17]. It is widely known that autophagy defects contribute to neurodegenerative diseases, as evidenced by the accumulation of undigested autophagic vesicles in the cytoplasm of surviving neurons. As a result, it has been reported how autophagosomes accumulate in many brain diseases (including severe AD and animal models), indicative of a slowing lysosomal clearance rate. Several neurodegenerative diseases, such as PD and Lewy body dementia (LBD), have been linked to autophagy dysfunction [18]. Recent studies have correlated autophagy to the pathophysiology of familial and idiopathic PD, and upregulating autophagy is an effective treatment for PD [17]. The primary regulator of the autophagy-lysosomal pathway (ALP), transcription factor EB (TFEB), regulates the expression of genes necessary for the development of lysosomes and autophagosomes [47, 48]. It has been demonstrated that the phosphorylated form of TFEB preferentially interacts with the chaperone-dependent E3 ubiquitin ligase STUB1, resulting in ubiquitination and subsequent degradation via the ubiquitin-proteasome pathway [49]. The nuclear export of TFEB is controlled by mTOR-dependent phosphorylation [50]. Due to an increase in the accumulation of LC3, ubiquitinated proteins, autophagosomes, and substrates, own modulation of TFEB significantly decreased ALP activity in rats with permanent middle cerebral artery blockage [51]. Numerous studies have suggested that TFEB is a promising therapeutic target for PD [52].

The mammalian target of rapamycin (mTOR) is the primary regulator of autophagy in macrophages [53]. Multiple signaling routes converge in the mTOR signaling pathway, which has recently become the most-studied signaling system due to its influence on autophagy via both upstream and downstream signal transductions [54–56]. Inhibiting mTOR stimulates autophagy and increases protein degradation through lysosomes [18]. Inhibiting p-mTOR can activate the autophagy process [57]. The dysfunction of any of the autophagy processes may damage the autophagy-lysosomal pathway [18]. The protein 1 light chain, 3 beta (LC3), which attaches to microtubules, plays an important function in autophagy by assisting in the closure of autophagosomes once they have formed [58]. The LC3-II/LC3-I expression ratio may be utilized as a proxy for autophagy activity because LC3-I becomes LC3-II during this process [59]. P62, an autophagy substrate, is increased when autophagy is suppressed but downregulated when induced [60, 61].

Also, plumbagin blocks Akt activation and downstream targets, reducing the phosphorylation of two mammalian targets downstream of mTOR [62]. Plumbagin inhibits the phosphatidylinositol 3-kinase (PI3K)/protein kinase B (Akt) signaling pathway. Studies have shown that plumbagin may activate autophagy in a dose-dependent manner, block the G2/M phase of cells, raise ROS levels, and inhibit the PI3K/Akt/mTOR pathway by decreasing Akt and mTOR phosphorylation [63]. These findings imply that plumbagin may promote autophagy in cells. Plumbagin can reduce the increase in p-mTOR protein in the substantia nigra of PD mice models, enhance autophagy, and expedite the clearance of autophagy substrates p62, thereby having a neuroprotective role. Furthermore, plumbagin antagonized MPP+ damage to SH-SY5Y and PC12 cells with improved autophagy.

Many studies on the anti-PD of active ingredients have been reported, and exploring the anti-PD effect of plant-active ingredients is a valuable research direction. Chlorogenic acid has demonstrated neuroprotection in MPTP-intoxicated rats through antioxidant and anti-inflammatory properties, as well as apoptotic death of DA neurons caused by mitochondrial dysfunction [64, 65]. In mice exposed to rotenone, ursolic acid reduced p62 and ubiquitin-aggregated proteins, improved autophagic clearance, and mitigated the loss of DA neurons [66]. Ursolic acid administration inhibited the neuroinflammation induced by MPTP, mainly reflected by reducing the expression of the ionic calcium-binding adaptor molecule 1 (Iba1) and TNF-*α* and inhibiting the nuclear translocation of NF-*κ*B. [67]. *Mucuna pruriens* administration significantly decreased glial fibrillary acidic protein (GFAP), iNOS, intercellular cell adhesion molecule (ICAM), and TNF-*α* inflammatory parameters in MPTP-induced Parkinson's disease (PD) animals. It also inhibited NF-*κ*B activation and increased p-Akt1 activity. Additionally, *Mucuna pruriens* demonstrated substantial antioxidant potential by inhibiting lipid peroxidation and nitrite [68]. *Mucuna pruriens* reduces iNOS expression in paraquat-induced PD mouse [69]. Moreover, *Withania somnifera* has shown a significant improvement in movement disorders and dopaminergic neuroprotection, paraquat-induced Parkinsonism, and downregulation of iNOS and Bax and induction of Bcl-2 protein expression in Maneb [70, 71]. Hopefully, more active ingredients for PD will be discovered in the future.

## 5. Conclusions

In conclusion, this work demonstrated that plumbagin inhibits PD in both cell and animal models. Additionally, plumbagin inhibits antineuroinflammation, induces autophagy, and accelerates the clearance of autophagy substrates, all of which contribute to its anti-PD effects *in vivo* and *in vitro*.

## Figures and Tables

**Table 1:** The primer sequences utilized in the present study.

Primer name	Primer sequence
TNF–*α*	Forward 5′ - CGTCGTAGCAAACCACCAAG - 3′
	Reverse 5′ - GACAAGGTACAACCCATCGG - 3′
IL–1*β*	Forward 5′ - AAGAAGAGCCCATCCTCTGTG - 3′
	Reverse 5′ - TGTTCATCTCGGAGCCTGTAG - 3′
IL–6	Forward 5′ - TTGGGACTGATGCTGGTGAC - 3′
	Reverse 5′ - GTGGTATAGACAGGTCTGTTGGG - 3′
GAPDH	Forward 5′ - GGTTGTCTCCTGCGACTTCA - 3′
	Reverse 5′ - TGGTCCAGGGTTTCTTACTCC - 3′

## Data Availability

The data supporting the findings of this study are available from the corresponding authors upon reasonable request.

## Abbreviations

PL: Plumbagin
MPTP: 1-Methyl-4-phenyl-1,2,3,6-tetrahydropyridine
TLR: Toll-like receptor
TNF-*α*: Tumor necrosis factor-alpha
NF-*κ*B: Nuclear factor kappa B
IL-6: Interleukin-6
IL-1*β*: Interleukin 1 beta
LPS: Lipopolysaccharide
MPP+: 1-Methyl-4-phenyl-pyridine ion
LC3: Microtubule-associated protein 1 light chain 3 beta
p-mTOR: Phosphorylated mammalian target of rapamycin
mTOR: Mammalian target of rapamycin
AD: Alzheimer's disease
DA: Dopamine
SNpc: Substantia nigra pars compacta
LBs: Lewy bodies
ROS: Reactive oxygen species
PI3K: Phosphatidyl 3-kinase
Akt: Protein kinase B.

## Supplementary Materials

Supplementary Materials Supplementary Figure S1: the expression of the *α*-synuclein protein in PD mice induced by MPTP or MPTP/probenecid. (A) Western blotting analysis of *α*-synuclein in mice treated with the normal saline and MPTP. (B) Quantitative data of the *α*-synuclein protein levels in each mice group. (C) Analysis of *α*-synuclein by Western blotting in mice administered MPTP and normal saline. (D) Quantitative data of the *α*-synuclein protein levels in each mice group. Data are expressed as the mean ± SEM (*n* = 3). ^∗∗^*P* < 0.01 and ^∗^*P* < 0.05. Supplementary Figure S2: plumbagin suppressed the expression of *α*-synuclein in PD mice induced by MPTP or MPTP/probenecid. (A) Western blotting analysis of *α*-synuclein in mice treated with the vehicle, MPTP, plumbagin+MPTP, and plumbagin. (B) Quantitative data of the *α*-synuclein protein levels in each mice group. (C) Analysis of *α*-synuclein by Western blotting in mice undergoing the following treatments: vehicle, MPTP/probenecid, plumbagin+MPTP/probenecid, and plumbagin. (D) Quantitative data of the *α*-synuclein protein levels in each mice group. Data are presented as the mean ± SEM (*n* = 3). ^∗∗∗^*P* < 0.001, ^∗∗^*P* < 0.01, and ^∗^*P* < 0.05.
