# Supplementary material for: Inhibition of the TLR/NF-κB Signaling Pathway and Improvement of Autophagy Mediates Neuroprotective Effects of Plumbagin in Parkinson's Disease
Source: Oxid Med Cell Longev. 2022 Dec 22;2022:1837278. doi: 10.1155/2022/1837278 (PMC9800084; doi:10.1155/2022/1837278)
Supplement: Supplementary Materials — Supplementary Figure S1: the expression of the α-synuclein protein in PD mice induced by MPTP or MPTP/probenecid. (A) Western blotting analysis of α-synuclein in mice treated with the normal saline and MPTP. (B) Quantitative data of the α-synuclein protein levels in each mice group. (C) Analysis of α-synuclein by Western blotting in mice administered MPTP and normal saline. (D) Quantitative data of the α-synuclein protein levels in each mice group. Data are expressed as the mean ± SEM (n = 3). ∗∗P < 0.01 and ∗P < 0.05. Supplementary Figure S2: plumbagin suppressed the expression of α-synuclein in PD mice induced by MPTP or MPTP/probenecid. (A) Western blotting analysis of α-synuclein in mice treated with the vehicle, MPTP, plumbagin+MPTP, and plumbagin. (B) Quantitative data of the α-synuclein protein levels in each mice group. (C) Analysis of α-synuclein by Western blotting in mice undergoing the following treatments: vehicle, MPTP/probenecid, plumbagin+MPTP/probenecid, and plumbagin. (D) Quantitative data of the α-synuclein protein levels in each mice group. Data are presented as the mean ± SEM (n = 3). ∗∗∗P < 0.001, ∗∗P < 0.01, and ∗P < 0.05. [file 1837278.f1.docx]

**Supplementary Files**


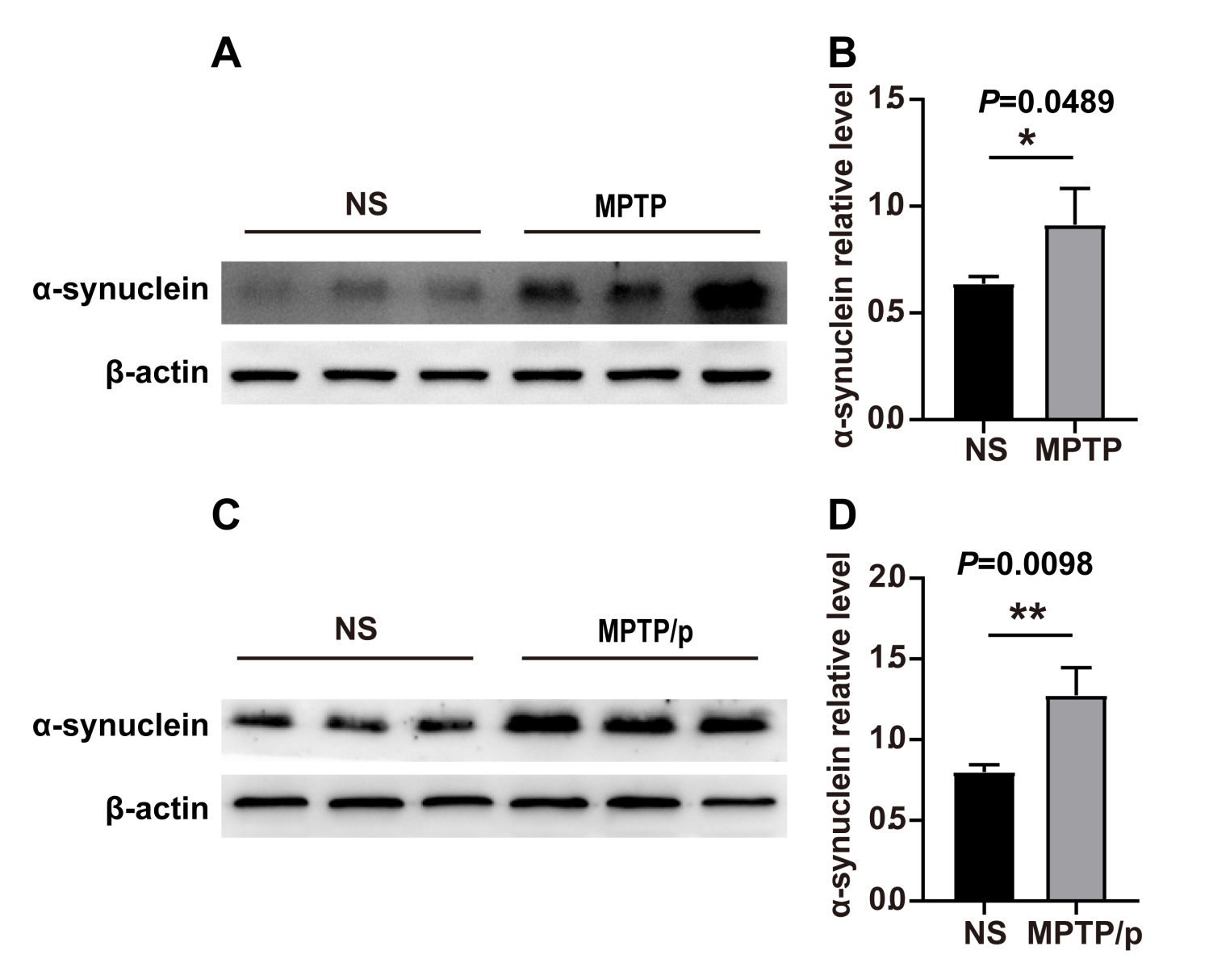


**Supplementary Figure S1: The expression of the α-synuclein protein in PD mice induced by MPTP or MPTP/probenecid.** (A) Western blotting analysis of α-synuclein in mice treated with the normal saline and MPTP. (B) Quantitative data of the α-synuclein protein levels in each mice group. (C) Analysis of α-synuclein by Western blotting in mice administered MPTP and normal saline. (D) Quantitative data of the α-synuclein protein levels in each mice group. Data are expressed as the mean ±SEM (n = 3). ***P* < 0.01, **P* < 0.05.


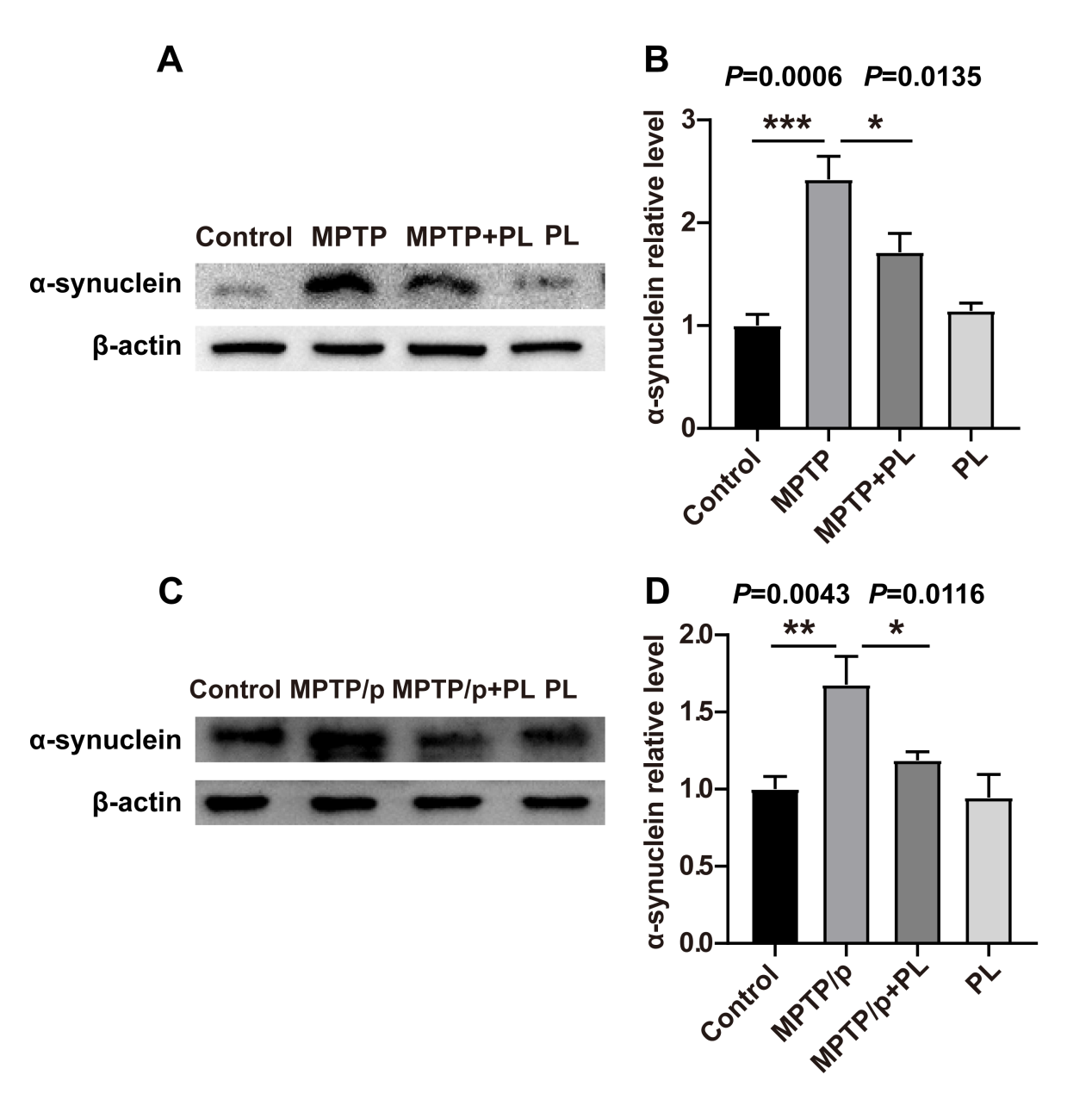


**Supplementary Figure S2: Plumbagin suppressed the expression of α-synuclein in PD mice induced by MPTP or MPTP/probenecid.** (A) Western blotting analysis of α-synuclein in mice treated with the vehicle, MPTP, plumbagin+MPTP, and Plumbagin. (B) Quantitative data of the α-synuclein protein levels in each mice group. (C) Analysis of α-synuclein by Western blotting in mice undergoing the following treatments: vehicle, MPTP/probenecid, plumbagin+MPTP/probenecid, and Plumbagin. (D) Quantitative data of the α-synuclein protein levels in each mice group. Data are presented as the mean ±SEM (n = 3). ****P* ＜ 0.001, ** *P* ＜ 0.01, * *P* ＜ 0.05.
